# A Numerical and Experimental Analysis of the Mechanical Behavior of the Aluminum Beverage Can with Internal Varnish Layers during Axial Load Force Testing

**DOI:** 10.3390/ma16196603

**Published:** 2023-10-09

**Authors:** Przemysław Wędrychowicz, Piotr Kustra, Andrij Milenin

**Affiliations:** Faculty of Metals Engineering and Industrial Computer Science, AGH University of Science and Technology, Mickiewicza 30 av., 30-059 Kraków, Poland; wedrycho@agh.edu.pl (P.W.); milenin@agh.edu.pl (A.M.)

**Keywords:** 3104 H19, beverage can, internal varnish, axial load force

## Abstract

This article presents a numerical and experimental investigation into the impact of can wall thickness and the internal varnish layer thickness on the results of an axial load force test. This study also shows the levels of thermal stresses that emerge after the drying of varnish coating, and how they affect the results of the axial load force test. This research involves the development of suitable numerical models and the experimental acquisition of stress–deformation relationships for the both can material, aluminum, and the varnish. The numerical simulation of the axial load force test has been verified through experimental tests, with a resulting difference of 8.9% between the two sets of results. The findings highlight that changes in the can wall thickness have a more pronounced effect on test outcomes compared to variations in the varnish thickness. Specifically, an increase in the can wall thickness from 90 µm to 100 µm results in a substantial 116 N increase in the force required for a can to collapse. Nevertheless, the presence of a 5 µm varnish layer also contributes measurably, increasing the can’s collapse force by 21 N. These results offer valuable practical insights for manufacturers, enabling them to effectively optimize can strength characteristics.

## 1. Introduction

Aluminum cans are widely used in the food and beverage industry due to their outstanding properties such as being lightweight, durable, and 100% recyclable. In particular, beverage aluminum cans have become increasingly popular due to their convenience, portability, and shelf stability. Over the years, numerous studies have investigated the physical, chemical, and mechanical properties of aluminum cans to improve their design and performance. The production process is complex. In the production line, aluminum sheets and cans are moved at a speed of about 1800–3000 cans per minute, and are processed and formed on about 20 machines, until finally, the finished can is placed on a transport pallet. The entire production process from the aluminum sheet to the finished can takes about 45 min. This process includes stages of cold forming, the parameters of which affect the properties of the final product [1,2]. It all starts with aluminum ingots, which are heated and processed into thin sheets. These sheets are then cut into circular blanks that are larger than the final can’s diameter. Next comes the cupping process, where the blanks are formed during a drawing process, resulting in cup-shaped parts. The partially formed cans then go through a series of redrawing and ironing steps. In these steps, the cup diameter is reduced to the final can’s diameter and height. This process also reduces the thickness of the can walls, resulting in higher walls. Once the cans have reached their intended dimensions, they undergo a washing process to remove any excess material.

**Figure 1:**
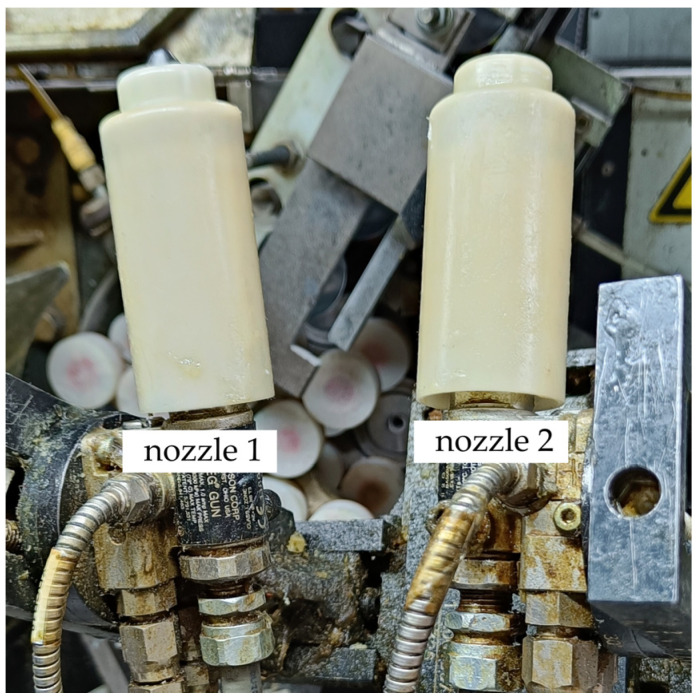
Internal spray application nozzles.

After washing, the cans are transported to a piece of specialized spray equipment that is used to apply the internal varnish layer. The spray equipment consists of a spray gun or nozzle (Figure 1) that atomizes the coating material into fine droplets and a system to control the spraying process. The equipment setup includes controls for adjusting the spray pattern, pressure, and flow rate to ensure a consistent and uniform coating application. The cans are loaded onto a rotating or spinning mechanism that holds the cans securely in place during the spraying process. The spinning action helps to evenly distribute the coating material across the interior surface of the cans. As the cans rotate, the spray gun or nozzle is positioned inside the can. The coating material is then sprayed in a controlled manner to ensure an even coverage. The fine droplets of the coating material adhere to the interior surface of the can, creating a uniform and protective layer.

After the coating is applied, the cans move to a curing stage. Curing involves subjecting the coated cans to a controlled environment in a so-called IBO oven, where the coating material chemically reacts and solidifies in temperatures reaching about 200 °C. The curing process may involve specific temperature and time parameters that are determined based on the type of the coating material being used.

The optimal strength of aluminum beverage cans performs a crucial role in the beverage can industry. An excessively strong can results in an increased material consumption, while a can with strength below critical levels is unacceptable. This is why the final quality parameters of the can, especially the values of the force achieved in the axial load force test (ALF) is important. The results of the ALF test may be influenced not only by the stages of cold plastic forming [3] or the tool geometry [4] but also by intermediate stages such as the washing, application, and drying of the internal varnish, the applying and drying of lithography, and the path of the can through the entire production line. It has been found that all stages of production have an impact on the material from which the can is made and on the geometric parameters of the can, which translates into the results of the ALF tests [5].

Many studies aim to investigate and understand the influence of thin films on the mechanical properties of bulk materials. These gradient materials exhibit previously unprecedented characteristics, providing new possibilities for their application [6,7]. The literature currently lacks comprehensive quantitative data regarding how the thickness of the internal varnish layer, in conjunction with the can wall thickness, affects the mechanical properties of the finished can, particularly in relation to the results of the ALF test [5]. This research gap underscores the significance of our study, as it seeks to fill this void of knowledge by providing quantitative insights into these critical factors and their influence on a can’s strength. Therefore, the primary objective of this article is to present and thoroughly analyze the research findings concerning the influence of the internal varnish layer’s thickness and the can wall thickness on the performance of cans during the ALF test. By doing so, this study aims to contribute valuable insights to the field, aiding in the advancement of the beverage can manufacturing processes.

## 2. Materials and Methods

### 2.1. Aluminum Alloy 3104 H19

Aluminum alloy 3104 is a commonly employed material in the manufacture of aluminum beverage cans. Belonging to the 3000 series of aluminum alloys, it is recognized for its remarkable formability, corrosion resistance, and moderate strength [8]. Specifically designed to accommodate deep drawing processes, aluminum alloy 3104 is well suited for crafting beverage cans [9]. This alloy’s composition primarily comprises aluminum as the foundational metal, supplemented by small quantities of manganese (Mn) and magnesium (Mg). These alloying elements perform a pivotal role in enhancing the alloy’s strength and formability [10].

What sets aluminum alloy 3104 apart is its exceptional formability, a crucial attribute that makes it suitable for shaping intricate beverage can forms without encountering significant cracking or tearing issues. Furthermore, the natural corrosion resistance of aluminum, coupled with the added protection from manganese and magnesium, endows aluminum alloy 3104 with robust resistance to environmental factors and the contents of the beverage cans. Although not the strongest among aluminum alloys, its strength-to-weight ratio is more than sufficient for meeting the structural demands of beverage cans. This attribute is particularly valuable in applications where weight considerations are paramount. In conclusion, the unique blend of formability, corrosion resistance, and moderate strength makes aluminum alloy 3104 an optimal choice for the fabrication of aluminum beverage cans. Its characteristics contribute to the creation of lightweight, durable cans that effectively preserve the quality and integrity of the beverages they encase.

### 2.2. Internal Varnish

Internal varnish, also known as internal coating, is an essential element in the beverage can manufacturing process. This specialized coating serves multiple purposes to ensure the quality of both the beverage and the can itself [11]. The primary objectives of internal varnishing in beverage can manufacturing are twofold: preservation and protection. The varnish is applied to the interior surface of the can, creating a barrier between the beverage and the metal of the can. This barrier prevents any undesirable interactions between the beverage and the can, preserving the taste and quality of the drink. This is particularly important for beverages that are prone to flavor alterations or contaminations when in contact with metal, such as carbonated drinks and fruit juices. Furthermore, internal varnish acts as a corrosion-resistant layer. By shielding the metal interior from direct contact with the beverage, it prevents corrosion, which could compromise the structural integrity of the can [12]. This corrosion resistance is vital for ensuring the safety of the beverage and maintaining the can’s durability.

For applications involving food contact within metal cans, the frequently utilized resins encompass epoxy, phenolic, polyester, acrylic, vinyl, oleoresins, and their various iterations [13]. It is important to note that there is not a single resin type that universally caters to all food types; rather, each resin is specifically tailored for particular categories of food. As an example, epoxy acrylic resins that are cross-linked with amino resins find common usage in beverage cans. On the other hand, white aluminum-pigmented epoxy resins are better suited for preserving the quality of fruits and vegetables. Meanwhile, the utilization of phenolic and epoxy–phenolic blends, often incorporating an aluminum pigment, is prevalent for safeguarding sulfur-bearing fish and meat products [14].

In the process of can manufacturing, the metal undergoes various stresses, some of which alter the cylindrical shape of the can (such as flanging for attaching can ends), while others enhance the axial and dome strength of the can. Furthermore, the top of the can is often necked to reduce the lid’s size and the can’s headspace. Coatings that are applied before the necking process must possess the ability to endure these operations.

An example of an internal coating that is used in the production of aluminum beverage cans is Aqualure 900. This is a water-based, modified epoxy lacquer type used for the interior protection of 2-piece beverage cans. This coating is characterized by a viscosity of 14–16 s FORD 4 at 25 °C and a solids content of 18.5–19.5%. After the curing process, the generated layer of varnish has a density of 1.27 g/m^3^.

In the 1950s, epoxy resins were introduced as coatings for aluminum and steel cans. Their stability, protective function, and technical properties made them the most commonly used coating material. Most epoxy coatings are synthesized from bisphenol A (BPA, CAS 80-05-7) and epichlorohydrin, forming bisphenol A-diglycidyl ether epoxy resins. Many different blends of epoxy coatings were developed, with epoxy–phenolic coatings being the most important subgroup. Other blended resins are, e.g., epoxy amines, acrylates, and anhydrides.

### 2.3. Material Testing

To examine the influence of the internal coating applied to the inner side of a can on the strength of the can in an axial load test, it was decided to prepare a numerical model of this test for cans both with and without an internal coating layer. The first step in building the numerical model was to determine the boundary conditions of the test and to study the aluminum alloy 3104 H19, from which the can is made, and the mechanical properties of the internal coating. To confirm the correctness of the basic numerical model of the ALF test, the simulation results were compared with the experimental tests. The ALF tests of the can were performed on the Zwick250 strength testing machine, produced by The ZwickRoel Group (https://www.zwickroell.com, accessed on 19 July 2023).

To evaluate the characteristics of the internal varnish, essential to conducting the finite element method (FEM) simulations, the mechanical parameters of the internal varnish were needed. To carry out the uniaxial compression test, cylindrical samples of the internal varnish were prepared.

**Figure 2:**
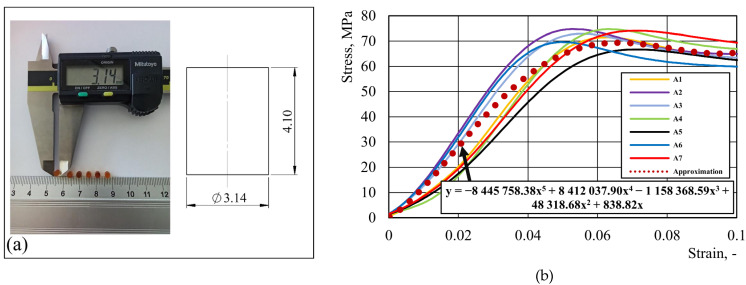
Internal varnish: (**a**) sample for the uniaxial compression test; (**b**) results from seven uniaxial compression test.

The process of creating epoxy-based varnish samples started by preparing an appropriately thick layer of varnish. Subsequently, numerical milling was employed to shape the varnish into cylinders with a diameter to height ratio of 1.2 (Figure 2a). For the uniaxial compression experiments, a total of seven varnish samples were subjected to testing using a Zwick250 machine, employing a strain rate of 0.08 s^−1^. The averaged results are YS = 67.49 ± 15.87 MPa and UTS = 71.00 ± 3.29 MPa, and the individual tests are presented in Figure 2b. The received data were used in the numerical model of ALF test procedure.

**Figure 3:**
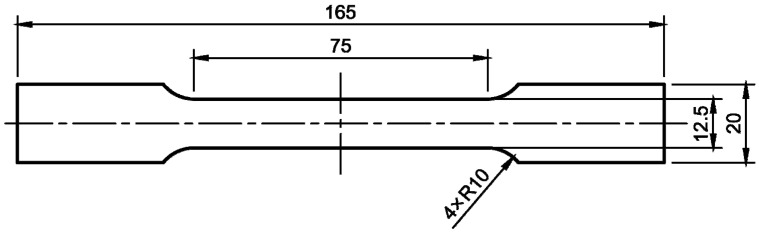
Sample according to PN-EN 10002-1 + AC1 used in the uniaxial tensile test of aluminum alloy 3104 H19.

The second tested material was an aluminum alloy 3104 H19 used for making cans. Axial tensile tests were conducted using a Zwick Z250 testing machine, all of which were performed under ambient room temperature conditions. In this test, the standard specimens prepared according to PN-EN 10002-1 + AC1 [15], shown in Figure 3 were used. The testing protocol included applying a strain rate of 0.005 s^−1^. Throughout these tests, the specimens were progressively stretched until reaching the point of failure.

**Figure 4:**
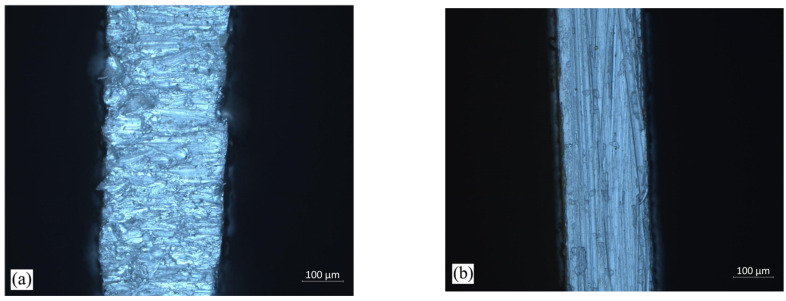
Tensile specimen edge picture after: (**a**) water jet cut; (**b**) milling.

**Figure 5:**
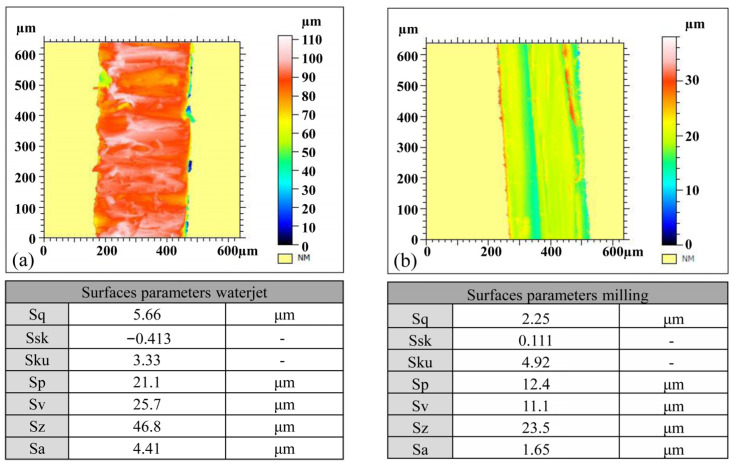
Surface parameters according to ISO 25178 for: (**a**) water jet cut specimen; (**b**) milling specimen.

**Figure 6:**
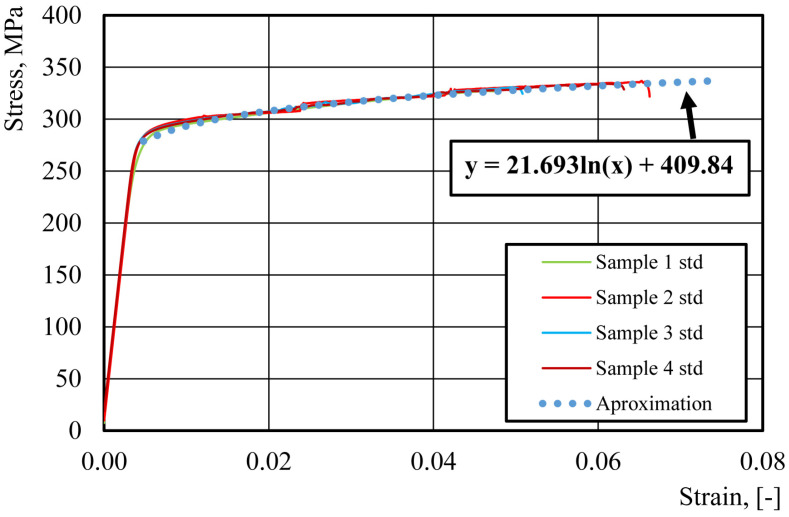
Tensile test results of aluminum alloy 3104 H19 (PN-EN 10002-1 + AC1 standard sample).

The results of tensile tests on thin materials can be influenced by the quality of the lateral surface of the samples. A high surface roughness can result in stress concentration on the surface, potentially distorting the measurement results. Therefore, prior to cutting the samples for uniaxial tensile tests on the aluminum alloy, a decision was made to assess the quality of the sample edges using two different cutting technologies. One of these was water jet cutting technology, and the other was milling. The advantage of these technologies is the lack of influence of the temperature generated during cutting on the sample material, as is the case when cutting with a laser or plasma. Observations under an optical microscope of the edges of the samples that were cut using the water jet and milling technologies allowed us to assess their quality (Figure 4). The roughness analysis of the cut surface was performed according to the standard ISO 25178 [16]. These studies showed that the milled samples had significantly less roughness (Figure 5). Therefore, in further studies, all samples were prepared via milling. The average value of the four tensile tests are YS = 293 ± 1.12 MPa, UTS = 328 ± 1.94 MPa, A = 6.1 ± 0.19%. The individual tests are shown in Figure 6.

### 2.4. Numerical Models

**Figure 7:**
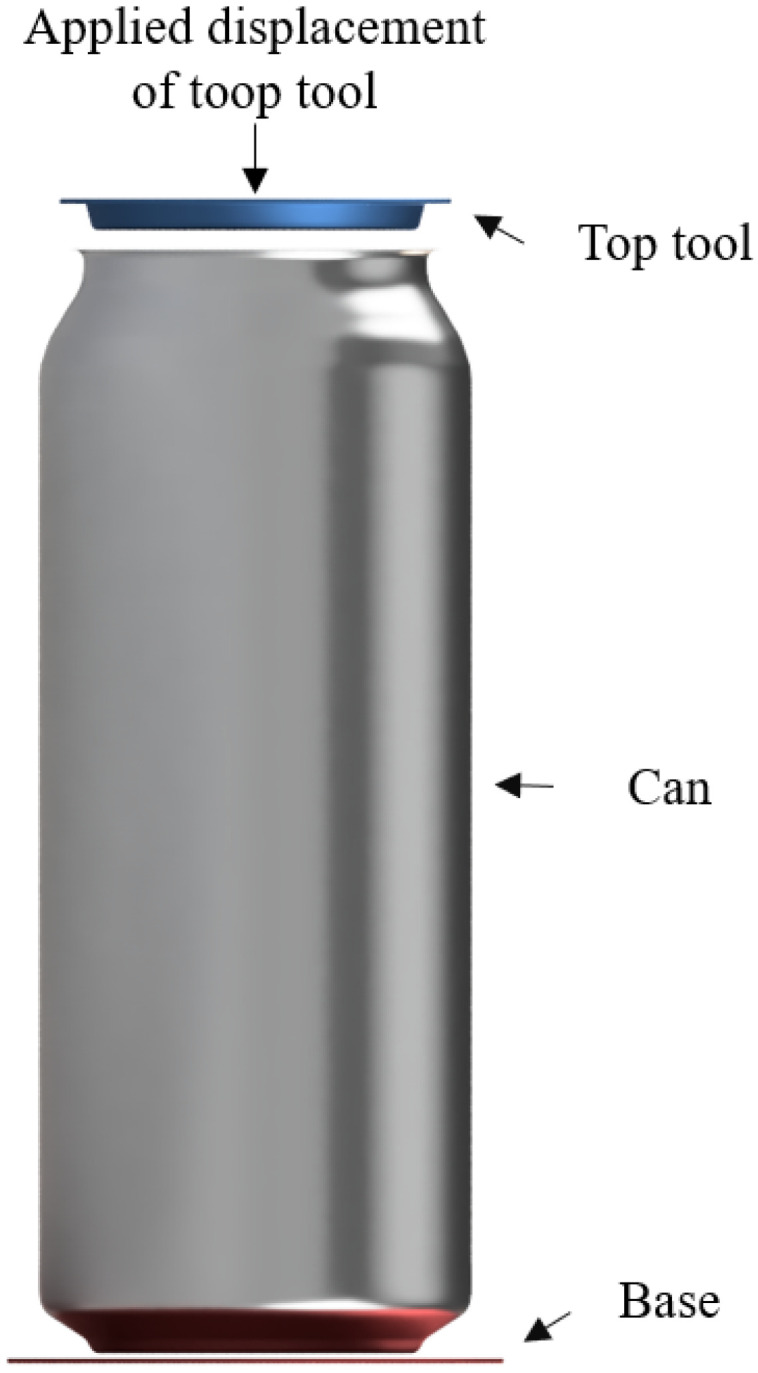
Axial load force test boundary condition visualization.

The next stage of this study involved the preparation of a numerical model for the ALF test. As outlined in the existing literature [5], a multitude of factors perform a significant role in influencing the force values that are obtained from ALF testing, as well as in determining the failure mode exhibited by the can during this particular assessment. Surprisingly, despite these investigations, prior research has not explored the potential impact of internal varnish coatings. To comprehensively evaluate the ramifications of internal varnish coatings on the outcomes of the ALF test, a series of finite element method (FEM) models were developed with a boundary condition, as presented in Figure 7. These models simulate the behavior of the beverage can both with and without the presence of a varnish coating layer.

To FEM simulate the behavior of the can body, LS-Dyna, a commercial solver, was employed in conjunction with the preprocessor LSPrepost. The initial geometry used for simulating the tests was obtained from a can-forming simulation that is well documented in [1]. It was crucial to employ these models because the aforementioned article elaborates on how the thickness of the can’s wall varies along its edges due to the forming processes involved.

The flow stress of the aluminum alloy of the can’s body was incorporated into LS-Dyna, based on the tensile test results. For both the can body aluminum alloy and the epoxy-based internal varnish, LS-Dyna material model 024 [17], which is based on J2 plasticity, was applied. In relation to the epoxy-based varnish material, based on the test results presented in Figure 2, the following parameters were included in the material model: Young’s modulus E = 1.1 GPa, yield strength YS = 67.49 MPa, and Poisson’s ratio ν = 0.35. For aluminum alloy 3104 H19, based on the test results presented in Figure 6, the following material parameters were defined: Young’s modulus E = 69.0 GPa, yield strength YS = 293.0 MPa, and Poisson’s ratio ν = 0.33. To specify the elastic–plastic domain for the internal varnish and aluminum alloy, the tabulated data that were extracted from the internal varnish compression test results (Figure 2b) and the aluminum alloy tensile test results (Figure 6) were used.

**Figure 8:**
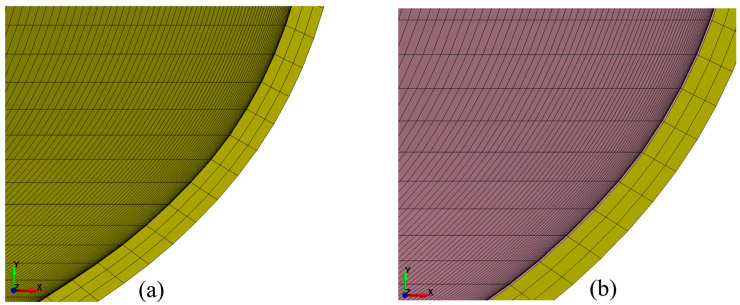
Fragment of cross-sectional views of the numerical model for the can: (**a**) without the internal varnish layer; (**b**) with the internal 5 μm varnish layer.

Before conducting numerical simulations of the ALF test, a simulation of the cooling stage after the varnish hardening process at around 200 °C was performed. The aim of this simulation was to verify the level and distribution of thermal stresses that were generated during the cooling of the aluminum and internal varnish from 200 to 20 °C, and to include this stage’s impact in the ALF test. The model of the can and varnish with the stresses from the cooling process, resulting from different coefficients of linear expansion of the aluminum and internal varnish, respectively, 2.3 × 10^−7^ 1/°C and 7.0 × 10^−7^ 1/°C, was then used in the ALF test simulations. The construction of numerical models for the can was defined as a 3D model with solid elements, as shown in Figure 8. The created model of the can without an internal varnish layer comprises 4,787,376 elements, and for the model with the layer of internal varnish, 6,383,168 elements were used.

## 3. Results

**Figure 9:**
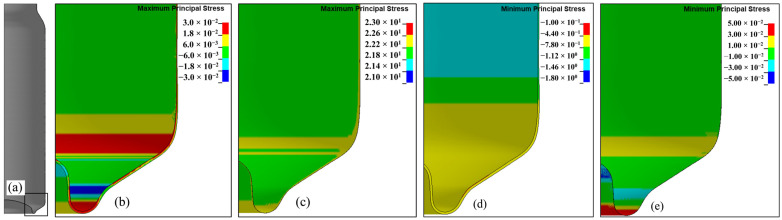
Results of the cooling simulation from 200 °C to 20 °C; (**a**) whole can body picture; (**b**) aluminum—max principal stress values in MPa; (**c**) varnish—max principal stress values in MPa, (**d**) aluminum—min principal stress values in MPa; (**e**) varnish—min principal stress values in MPa.

The results of the cooling simulations included the distribution of residual stresses in the internal coating layer and the aluminum can wall after the cooling stage from 200 to 20 °C. As expected, given that the internal coating has a coefficient of thermal expansion more than twice that of aluminum, the compressive stresses predominantly affected the can wall. The distribution of these compressive stresses at a selected point in the can is depicted in Figure 9. Analyzing the distribution of principal stresses in the coating layer, it can be concluded that it is subjected to the action of tensile stresses (Figure 9c), with a maximum value of approximately 22 MPa, while in the aluminum layer, principal stresses of approximately −1.43 MPa were created (Figure 9d).

**Figure 10:**
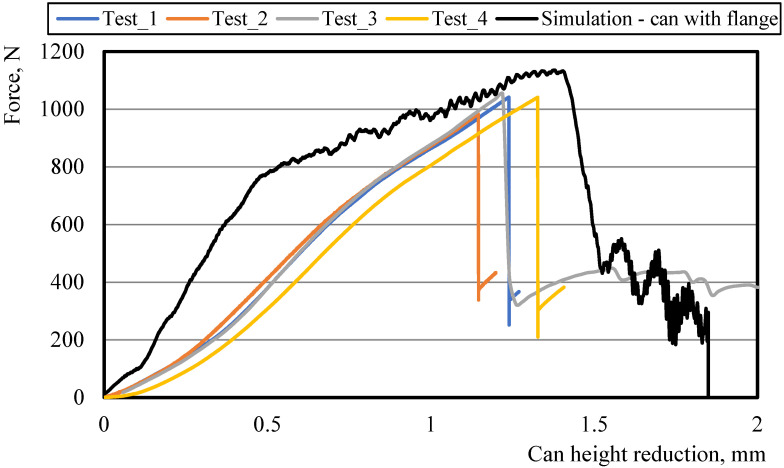
Comparison of simulation and experiment results in the axial load force test for a can with a 95 μm aluminum wall and a 5 μm internal varnish layer.

As mentioned earlier, the cooling simulation results for the can were used as batch data in the ALF test simulations for the can with an internal varnish layer. The obtained numerical results for the ALF test of a can with an aluminum wall thickness of 95 μm and an internal varnish layer thickness of 5 μm were compared with the results of the axial compression experiment results of a can with the same parameters (the experiment was repeated five times). The charts of the force as a function of the upper clamping tool’s displacement for the numerical model and experimental tests have been presented in Figure 10.

**Figure 11:**
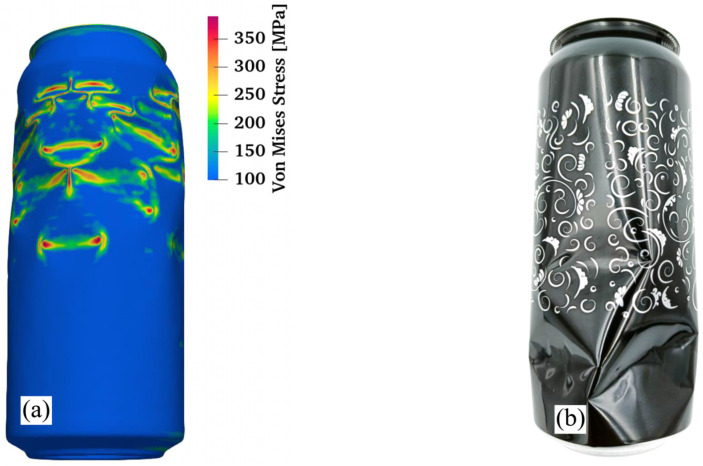
Failure mode of a beverage can for the ALF test comparison; (**a**) simulation; (**b**) experiment.

In production practice, the top edge of the can has a significant impact on the values of the force achieved in the ALF test. This is likely why the force value that was achieved in the numerical model is 8.9% higher than the averaged maximum force of 1031.28 ± 24.36 N that was obtained in laboratory tests. Other imperfections in the manufactured cans, such as circumferential thickness variation, can sidewall dents or other geometrical imperfections, or even imperfections of the testing equipment, can contribute to these differences. In Figure 11, there is a comparison of how the numerical model of the can looks after the ALF test and how the can looks after the experiment. In both cases, the damage to the can occurred on its sidewall.

In the subsequent phase of the research, it was imperative to investigate how the varnish layer impacts the outcomes of the ALF test. It was also decided to investigate the influence of the aluminum thickness on the ALF test results and whether the thermal stresses generated during the cooling process of the can from 200 °C to 20 °C have an impact on the ALF test results. For this purpose, appropriate numerical models were prepared and used in the ALF test simulations. The comparison of the models, together with the results of the conducted ALF test simulations, has been collected in Table 1.

Looking at the results of the ALF test simulations for models 1 to 6, we can see that varying the wall thickness and varnish thickness has an impact on the force that is required to deform the can. Models 1 to 3 have the same varnish thickness of 0 μm but varying wall thicknesses. As expected, an increase in the wall thickness generally results in a higher ALF force result. In this case, we see that Model 3, with the thickest wall, requires the highest force of 1178 N for the can to buckle.

**Figure 12:**
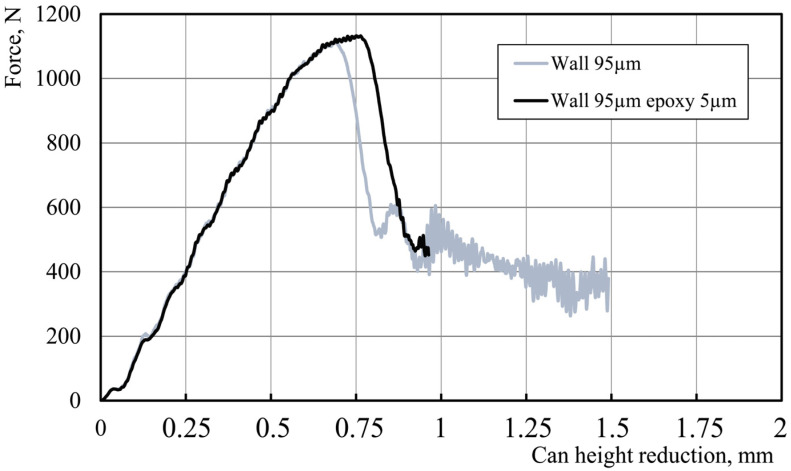
Comparison of the can body behavior under axial loading force for a can with a 5 μm varnish layer and a can without varnish (can models without flange).

Models 4 and 5 both have the same wall thickness of 95 μm but varying varnish thicknesses. We can see that the varnish thickness has a smaller impact on the ALF force requirement than the wall thickness. Model 4, with a varnish thickness of 10 μm, results in a slightly higher force than Model 5, with a varnish thickness of 5 μm. Model 6 is unique in that it includes thermal residual stresses in addition to the physical properties of the can. However, its ALF force result of 1133 N is similar to that of Models 4 and 5, suggesting that the effect of thermal stresses are less significant than the effect of changes in the wall thickness and varnish thickness. Overall, these results suggest that the physical properties of the can, particularly the wall thickness, have the most significant impact on the ALF force results. The effect of the varnish thickness is also noticeable, while the effect of thermal stresses is comparatively small. When we look at the compression test results for the can with and without a varnish layer, and particularly at the results showing the force–displacement curve, we can say that in the initial phase of the test, the behavior of the can body, both with a varnish layer and without it, is the same (Figure 12). In both cases, the maximum can stiffness is equal to about 1738 N/mm.

**Figure 13:**
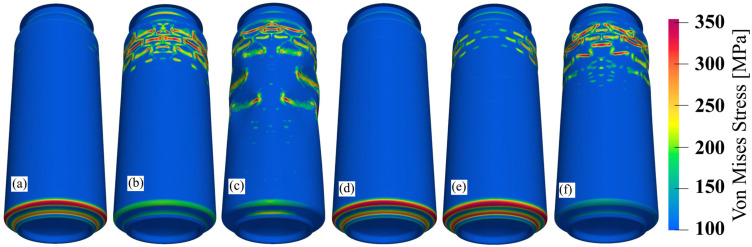
Von Mises stress distribution on the can: (**a**–**c**) model without varnish; (**d**–**f**) model with a 5 μm varnish layer; (**a**,**d**) before can sidewall buckling; (**b**,**e**) after can sidewall buckling; (**c**,**f**) after some time of can sidewall buckling.

Differences in the mechanical behavior can only be observed in the final phase of the test, when the influence of a 5 μm thick layer of varnish on the value of the force at which the sidewall of the can breaks is visible. The presented difference in the achieved level of the maximum force obtained in the ALF test is also noticeable in the lower levels of stress that were obtained for the can model both with a layer of varnish and without it at the same moment in the test, which ultimately results in a higher force of can destruction. Figure 13 show the stress levels that were obtained at the same moment during the ALF test for the can models both with and without an internal varnish layer.

**Figure 14:**
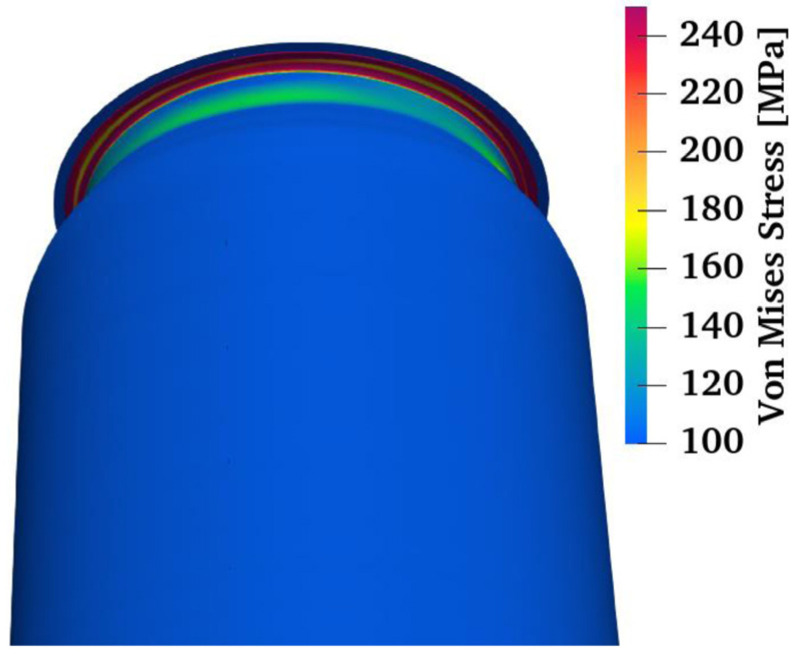
Can flange deformation region during the axial load force test.

During the analysis of the results of the ALF test, it was also observed that the force vs. top tool displacement curve in the numerical model differs from the curve obtained in the experiment (Figure 10). Nonlinearity was observed in the behavior of the can during this test. In observing the behavior of the can body and the stress distribution on the numerical model of the can during the ALF test, it was found that the flange of the can may have an impact on the nonlinearity in the can’s behavior (Figure 14).

**Figure 15:**
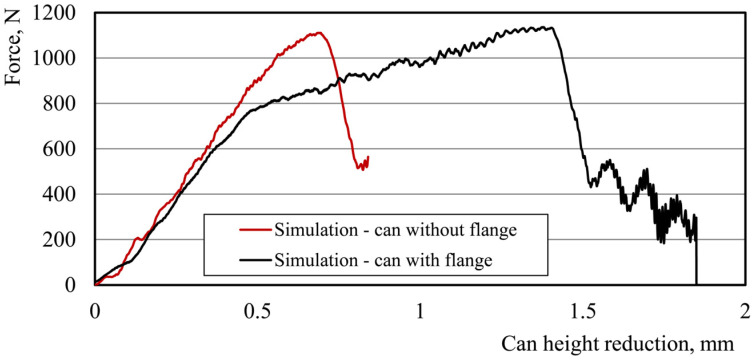
Simulation results of can body behavior under axial loading force for models with and without a flange.

At the beginning of the study, the flange of the can was omitted from the simulation, as it was assumed that it would not affect the results of the ALF test. To verify the impact of the can’s flange on its behavior during the ALF test, it was decided to simulate this test for a can without a flange. The results confirmed the hypothesis of the impact of the flange on the can’s behavior. As indicated in the simulation results, the flange does indeed influence the can’s behavior but does not have any impact on the axial force achieved in the ALF test (Figure 15).

## 4. Discussion

The main goal of this conducted research was to examine the influence of the internal varnish layer that is applied to an aluminum beverage can during the production process on the obtained ALF test force value. To do this, numerical models of cans with an internal varnish layer and without were created, and the results of the obtained forces for these models in ALF test simulations were compared. To prepare such models, it was necessary to first obtain material data for both the 3104 H19 aluminum alloy from which the can is made, as well as for the internal varnish. In order to investigate the mechanical properties of the aluminum alloy, plasticity tests were conducted using a Zwick 250 universal testing machine and standard specimens (Figure 3). To eliminate the impact of the heat that is generated during laser cutting, alternative methods such as water jet cutting or milling were considered for sample preparation. However, it was unknown which technology would provide better quality samples. Therefore, samples were prepared using both methods and their cutting quality was evaluated using an optical microscope and surface topography analysis software. An edge analysis of the samples revealed that those prepared via milling had edges of better quality. As sample edge quality can significantly affect the results of a uniaxial tensile test, samples with lower edge roughness parameters were selected. Subsequently, the preparation of internal varnish samples for an axial compression test was undertaken. The specimens’ preparation started with the creation of an appropriately thick layer of varnish, from which cylindrical samples with a diameter-to-height ratio of 1.2 could be milled. Using a CNC milling machine, the internal varnish was milled to create the required samples, which were later subjected to axial compression. The received results were used as the input data for building models of cans with and without a layer of coating. As the internal coating obtains its final strength parameters after the drying process at a temperature of about 200 °C, it was decided to also examine the influence of the stresses that are generated in the coating layer and aluminum can wall after the cooling stage to 20 °C. The results of the cooling analysis were used in one of the models of a can with a 5 μm coating layer and simulations of the ALF test. This allowed us to answer the question of how each of the parameters—the thickness of the aluminum wall, the thickness of the internal coating, and the thermal stresses that occur after the drying process—affect the results obtained in the ALF test.

Since aluminum cans are one of the most popular forms of beverage packaging, billions of these packages are produced worldwide every day [18]. That is why the technology of their production is constantly being developed and optimized [19]. The authors of the study of [5] examined the impact of imperfections in the geometry of a can, such as sidewall dents, varying thicknesses, and top-edge slants. They conducted physical tests on what are known as “bright cans”, meaning that the cans had no internal or external coating layers. As the presented research shows, the impact of the internal coating layer cannot be ignored when it comes to the values of axial force that are achieved in ALF testing, which, in the production process, is measured for cans with both an internal coating layer and external lithography. The authors of the study [20], which examined the influence of friction coefficient, can diameter, wall thickness, and punch speed on the formation of wrinkles during the necking process, did not include the internal layer of coating in their numerical models. As seen in the example of the ALF test, this can also have a noticeable impact on the occurrence of defects in necking process. The results of the conducted research are also significant from the point of view of potential modifications to the technology of aluminum beverage can production, for example, in the field of internal coating composition, and application and coating drying technology. Like other researchers who have attempted to join dissimilar materials to achieve new properties [21,22], modifications to the internal coating composition may also result in changes in application technology, drying technology, the thickness and distribution of the resulting layer, the mechanical parameters of the coating itself, and, as shown in the conducted research, it does not remain without an impact on the performance of the can. The observations made also provide the opportunity to optimize the existing technology, for example, to create a more uniform layer of paint or through a more precise application method, to mechanically strengthen selected areas of the can by applying a thicker layer of internal coating.

## 5. Conclusions

The key findings of this study, which provide practical insights for optimizing can strength, are as follows:

- The force value that was achieved in the reference numerical model is 8.9% higher than the averaged force of 1031.28 ± 24.36 N obtained in laboratory tests;
- The thickness of the aluminum can wall is the most influential factor in ALF test force variations;
- A 5 μm internal varnish layer increases the axial force in the ALF test by 21 N;
- Increasing the varnish layer by a further 5 μm, i.e., to a value of 10 μm, practically did not cause any further increase in the axial force achieved in the ALF test;
- Thermal stresses during cooling do not significantly affect the ALF test force;
- The can’s flange affects its behavior but not the ALF test’s axial force;
- These findings provide practical insights for optimizing cans’ strength and production technology;
- The aluminum alloy 3104 sheet samples prepared for plastometric testing using milling technology have a superior edge quality compared to samples prepared using water jet cutting.

## Figures and Tables

**Table 1:** Numerical model of the ALF simulation results summary.

Model	Wall Thickness (μm)	Varnish Thickness (μm)	ALF (N)
1	90	0	995
2	95	0	1111
3	100	0	1178
4	95	5	1132
5	95	10	1135
6 (with residual stresses)	95	5	1133

## Data Availability

Not applicable.
